# A novel personal cooling system (PCS) incorporated with phase change materials (PCMs) and ventilation fans

**DOI:** 10.1186/2046-7648-4-S1-A136

**Published:** 2015-09-14

**Authors:** Yehu Lu, Fanru Wei, Dandan Lai, Wen Shi, Faming Wang, Chuansi Gao

**Affiliations:** 1 Laboratory for Clothing Physiology and Ergonomics (LCPE), the National Engineering Laboratory for Modern Silk, Soochow University, Suzhou, China; 2 Department of Design Science, Lund University, Lund, Sweden

## Introduction

Heat stress has been considered as a common risk to impair work performance, which may lead to heat illnesses, work incidents, or even fatality. Over the past few decades, various types of personal cooling systems (PCSs) have been developed to mitigate heat strain, e.g., cooling vests with ice packs or phase change material (PCM) packs or frozen gel strips, garments with liquid cooling systems and garments with forced air ventilation. In this study, a novel personal cooling system (PCS) incorporated with both PCMs and ventilation fans was developed and its cooling efficiency was evaluated on a sweating manikin.

## Methods

A novel hybrid portable PCS incorporated with PCMs and ventilation fans was developed, which consisted of a long sleeve jacket and a pair of long pants. Four fans were embedded at the lower back and the lateral pelvis regions of the PCS. Twenty-four PCM packs were also incorporated into the PCS. Six PCM packs were located at the chest region, 8 packs at the back region, 4 packs at the upper arm regions and 6 packs at the front thigh regions. The melting temperature of the PCMs is 21 °C and the latent heat of fusion is 144 J.g^-1^. The total mass of PCM packs is 2064 g. A polyester T-shirt and briefs were worn under the PCS. A Newton type 34-zone sweating thermal manikin was used. A constant surface temperature of 34.0 °C was used to evaluate the cooling effect of PCS in the so-called isothermal condition (*T_manikin _= T_a _= T_r_*). The sweating rate was set at 1200 mL.hr^-1^.m^-2^. Four test scenarios were chosen: fans off with no PCMs (Fan-off, *i.e*., CONTROL), fans on with no PCMs (Fan-on), fans off with fully solidified PCMs (PCM+Fan-off), and fans on with fully solidified PCMs (PCM+Fan-on). Two hot environments were selected: hot humid (HH) and hot dry (HD). The relative humidity (rh) of the HH and HD conditions was 75 % and 28 %, respectively, and the mean (SD) air velocity was 0.4 (0.1) m.s^-1^.

## Results and discussion

**Figure 1:**
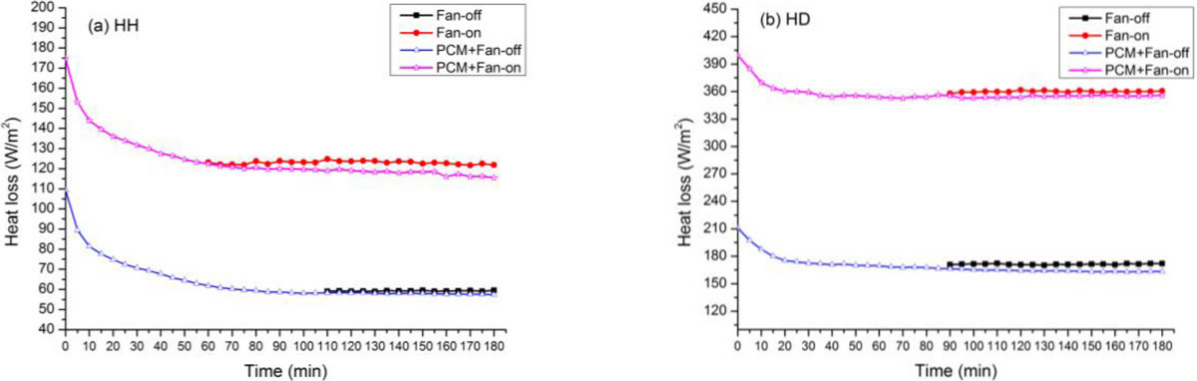
**Heat losses under four scenarios in HH (a) and HD (b) condition**.

Figure 1 shows the total heat loss under different scenarios in HH and HD condition, respectively. In both conditions, the heat loss of Fan-on was higher than that of Fan-off. The PCMs led to a higher manikin heat loss at the initial testing stage and it gradually decreases to a steady state. There was no significant heat loss difference between PCM+Fan-on and Fan-on when the PCMs were fully melted. Similarly, no heat loss difference was found between PCM+Fan-off and Fan-off. The PCM+Fan-on and Fan-on exhibited a higher heat loss than PCM+Fan-off. The PCMs provided about 90~110 min and 20~30 min cooling duration in HH and HD conditions, respectively.

## Conclusion

The results demonstrated that the portable PCS developed could be able to provide effective heat removal from a sweating manikin in two hot environments, with fan cooling being much more effective with a lower rh. The PCMs applied in the PCS provide effective cooling at the initial stage; as the manikin skin surface gradually gets wet, ventilation fans start to provide evaporative cooling.

